# A comparative analysis of sociodemographic and clinical characteristics of gambling disorder by sex among treatment-seeking individuals: A national sample from Türkiye

**DOI:** 10.1556/2006.2026.00083

**Published:** 2026-05-26

**Authors:** Yasin Kavla, Süleyman Agah Demirgül, Tuğba Bozdemir, Dilara Demircan, Büşra Yamanel-Özsoy, Hasan Durmuş, Eren Murat Dinçer, Gökhan Umut, Zsolt Demetrovics, Marc N. Potenza, Mehmet Dinç

**Affiliations:** 1 Department of Psychiatry, Cerrahpasa Medical Faculty, Istanbul University-Cerrahpasa, Istanbul, Türkiye; 2 Institute of Psychology, ELTE Eötvös Loránd University, Budapest, Hungary; 3 Doctoral School of Psychology, ELTE Eötvös Loránd University, Budapest, Hungary; 4 Trauma Intervention and Research Center/ Global and Regional Studies Center, Psychology Department, Necmettin Erbakan University, Konya, Türkiye; 5 Green Crescent Counselling Center, Türkiye; 6 Faculty of Medicine, Department of Public Health, Erciyes University, Kayseri, Türkiye; 7 Faculty of Arts and Sciences, Department of Psychology, Mimar Sinan Fine Arts University, Istanbul, Türkiye; 8 İstanbul Basaksehir Cam Sakura City Hospital, Istanbul, Türkiye; 9 Flinders University Institute for Mental Health and Wellbeing, College of Human Sciences and Culture, Flinders University, Bedford Park, SA, Australia; 10 Centre of Excellence in Responsible Gaming, University of Gibraltar, Gibraltar, Gibraltar; 11 Yale University School of Medicine, New Haven, USA; 12 Department of Psychology, Faculty of Economic Administrative and Social Sciences, Hasan Kalyoncu University, Gaziantep, Türkiye

**Keywords:** addictive behaviors, gambling, impulsive behaviors, gender and sex differences, compulsive behavior, risk-taking, behavioral addiction

## Abstract

**Background and Aims:**

Gambling disorder (GD) is a growing public health concern, with emerging evidence suggesting potential sex differences in clinical presentation, gambling preferences, and treatment-seeking patterns. Furthermore, non-Western countries/cultures and women's clinical populations are understudied. Therefore, this study aimed to examine sex-related sociodemographic characteristics, gambling behaviors, and psychiatric symptoms among treatment-seeking individuals with GD in Türkiye.

**Methods:**

Data from 12,505 treatment-seeking individuals who registered for Green Crescent Counselling Center between 2021 and 2024 were analyzed. A total of 330 women participants who met the inclusion criteria were identified and compared with a sample of 990 men participants (matched on age, marital status, and education). Sociodemographic and clinical features, gambling preferences, and psychiatric symptoms were compared between groups.

**Results:**

A notable increase in the number and proportion of women seeking-treatment for GD was observed between 2021 and 2024. Women reported beginning gambling at a later age and engaging in it for a shorter duration compared to men. They showed a distinct gambling pattern, with a strong preference for online casino games, whereas men primarily engaged in sports betting. Women also exhibited higher rates of co-occurring psychiatric concerns, including depression, anxiety, PTSD, along with greater impulsivity.

**Discussion and Conclusions:**

Our findings highlight meaningful sex-related differences in the clinical presentation and gambling behaviors of treatment-seeking individuals with GD. The sizable representation of women suggests the need for sex-sensitive prevention and intervention strategies. Tailored approaches that address co-occurring psychiatric concerns, gambling preferences, and speculative motivations, may enhance treatment outcomes and inform future service provision in the treatment of GD, particularly for women.

## Introduction

Gambling disorder (GD) is a behavioral addiction characterized by persistent and recurrent maladaptive patterns of betting that lead to clinically significant impairment or distress (M. Brand et al., 2025). According to the DSM-5, GD is classified in the category of “Substance-Related and Addictive Disorders,” sharing neurobiological and clinical features with substance use disorders (Potenza et al., 2019). Clinical features include chasing losses, tolerance, withdrawal, and impaired control (American Psychiatric Association, 2013). Globally, the prevalence of GD is estimated to range from 0.2 to 5%, with considerable variation depending on the population studied and the criteria used (Calado & Griffiths, 2016). When considering sex, women exhibit lower prevalence estimates of GD (Salonen et al., 2022; Zakiniaeiz & Potenza, 2018). In one study, the lifetime prevalence of GD was reported to be 3.0% for men and 1.2% for women (Wejbera et al., 2021). Multiple negative consequences have been associated with GD, including but not limited to financial difficulties, familial and occupational dysfunction, co-occurring psychiatric conditions, and suicidality, thereby underscoring the public health importance (Dellosa & Browne, 2024; Langham et al., 2015; Mestre-Bach et al., 2025; Wall, Marionneau, Lindqvist, & Molander, 2025; Wardle, Reith, Langham, & Rogers, 2019, Losaberidze et al., 2025). The extant literature highlights notable differences between men and women with GD with respect to sociodemographic characteristics, addiction characteristics, prognosis, psychological characteristics, and treatment-seeking behaviors, predominantly in the Western population (Gartner, Bickl, Härtl, Loy, & Häffner, 2022).

When it comes to specific sex differences in relation to GD. Multiple studies have indicated that, compared to women, men are more likely to engage in gambling and initiate gambling at younger ages (Baker-Frampton, 2023; Gartner et al., 2022; Ronzitti, Lutri, Smith, Clerici, & Bowden-Jones, 2016). Gambling is prevalent among men in social settings (land-based gambling) (Hing, Russell, Tolchard, & Nower, 2016; Kairouz, Paradis, Nadeau, Tovar, & Pousset, 2016). However, the recent rapid emergence of online gambling platforms has profoundly transformed the gambling environment, making it more accessible and appealing, perhaps particularly to young individuals and women (Corney & Davis, 2010; López-Torres, León-Quismondo, & Ibáñez, 2021; McCarthy, Thomas, Bellringer, & Cassidy, 2019; Wardle, 2017). Thus, there may be an increase in the number of women with GD (Hare, 2015). Among treatment-seeking individuals with GD, women are more likely than men to be unemployed (Ronzitti et al., 2016).

In general, men tend to engage in gambling for excitement and financial gain (Estévez, Macía, & Macía, 2023). Conversely, women's gambling may more frequently be driven by emotional distress, particularly as a mechanism to alleviate negative emotions (Estévez et al., 2023; McCormack, Shorter, & Griffiths, 2014). The type of gambling preferred also varies according to sex. Men tend to prefer strategic forms of gambling, such as sports betting and casino table games (Baenas et al., 2024; Jiménez-Murcia et al., 2020), while women tend to prefer non-strategic forms, such as electronic gambling machines (slots) and bingo (Baenas et al., 2024; McCormack et al., 2014). Men have been reported to exhibit higher levels of impulsivity, including limited premeditation (López-Torres et al., 2021; Wirkus, Czernecka, Bühringer, & Kräplin, 2024). In contrast, women have typically demonstrated more psychological distress and indebtedness (Hakansson & Widinghoff, 2020; McCormack et al., 2014).

A “telescoping effect,” characterized by a more rapid progression from non-problematic gambling to GD in women as compared to men, has been reported for GD, similar to other addictive disorders (Grant, Odlaug, & Mooney, 2012; Potenza et al., 2001; Ronzitti et al., 2016; Slutske, Piasecki, Deutsch, Statham, & Martin, 2015). A Swedish study of online gambling revealed that women experiencing excessive debt and financial distress may face levels of hardship that are comparable to or more severe than those experienced by men (Håkansson & Widinghoff, 2020).

The psychological characteristics of individuals with GD may show significant differences according to sex. Co-occurring alcohol or drug use disorders are more prevalent among men seeking treatment, while in women, mood and anxiety disorders are more commonly present. Furthermore, most women compared to men have other psychiatric diagnoses preceding their GD diagnosis (Estévez et al., 2023; Larsson & Håkansson, 2022; Miller, Mide, Arvidson, & Söderpalm Gordh, 2023; Ronzitti et al., 2016), whereas men have demonstrated cognitive distortions more frequently, such as the “illusion of control” and “overconfidence in luck,” which have been associated with GD severity (Jiménez-Murcia et al., 2020; Wirkus et al., 2024). Women frequently experience emotional dysregulation and difficulties in identifying and managing emotions, which can further exacerbate GD (Estévez et al., 2023; Sancho et al., 2021).

In addition to affective disorders (i.e., depression and anxiety), other most frequent conditions in relation to GD are post-traumatic stress disorder (PTSD), non-suicidal self-injury (NSSI), and suicidal behaviors. PTSD is a mental disorder that arises from exposure to traumatic events. Such traumatic experiences may result from sexual violence, natural disasters, or severe accidents (Kessler et al., 2017). The prevalence of PTSD in the general population has been reported at 8.3% (Koenen et al., 2017); however, among individuals with a history of GD, this prevalence ranges from 11 to 15% (Toneatto & Pillai, 2016). Notably, this rate is higher among treatment-seeking individuals, with prevalence estimates ranging from 17% to as high as 56% (Kilpatrick et al., 2013). NSSI is defined as the intentional destruction of one's bodily tissue without suicidal intent, encompassing behaviors such as cutting, hitting, and scratching (Nock, 2009). The prevalence of NSSI is reported to range from 11.5 to 33.8% (Mannekote Thippaiah et al., 2021), with women being at a higher risk than men. Additionally, a positive association between NSSI and GD has been documented in a previous comprehensive systematic review (Losaberidze et al., 2025). However, current literature still lacks information about NSSI among men and women in treatment-seeking individuals (Losaberidze et al., 2025) in non-Western cultures. Previous research has indicated that suicidal behaviors are more prevalent in countries characterized by low levels of social integration, which is common in Western cultures due to an individualistic lifestyle (Lester, 2024). Therefore, in non-Western cultures, suicide in relation to GD may not be as high as seen in Western cultures. In addition to psychological characteristics among different sexes in relation to GD, the cultural aspects of gambling behavior and psychopathological relationship with GD have also been mentioned as an important area of research, as previous study samples have mostly been from Western countries/cultures, limiting us from generalizing the findings to non-Western populations (Czakó et al., 2025).

Cultural differences may not only influence gambling behaviors and related psychopathology but also help-seeking behaviors, as stigmatization in relation to gambling may differ from culture to culture. For example, in many Asian (Chen & Mak, 2008; Gainsbury, Hing, & Suhonen, 2014) and Latin American cultures (Alegria et al., 2013), mental health issues are often perceived as a sign of weakness. In Arabic cultures, such issues are frequently regarded as a form of divine punishment (Karam, 2018). This stigmatization extends beyond individuals demonstrating addictive behaviors to include their immediate family members as well (Chee & Lui, 2021). In examining gambling behaviors, the ethical considerations associated with gambling are intricately linked to cultural context. For example, in Muslim cultures, gambling has historically been regarded as morally unacceptable, whereas it is not regarded as morally wrong to the same degree in Western culture (Chee & Lui, 2021). However, this stigmatization may lead to a delay in seeking treatment and greater psychopathology, along with significant impairments.

Despite barriers to treatment that both men and women face, the proportion of men seeking treatment for GD is higher than that of women in Western countries (Håkansson, Mårdhed, & Zaar, 2017; Miller et al., 2023). Gambling is generally perceived as a more acceptable activity for men, which may reduce the stigma associated with seeking treatment for men compared to women (Quigley, 2022). Women with GD often experience stigmatization, including anticipated stigmatization (Penfold, Nicklin, Chadwick, & Lloyd, 2024; Quigley, 2022), and may be subject to greater social pressure (Beaulac et al., 2017). Furthermore, an inverse relationship between GD severity and treatment seeking has been proposed (Braun, Ludwig, Sleczka, Bühringer, & Kraus, 2014); however, this inverse association may also be prevalent in non-Western cultures. Therefore, in countries such as Türkiye, individuals may experience high levels of psychopathology and significant impairments in relation to GD as a result of greater stigmatization.

Moreover, besides ethical viewpoints of the society, culture may also have an impact on the type of gambling that is preferred. For example, a previous study revealed that individuals in non-Western cultures, such as Arabic and Vietnamese, are more likely to engage in gambling behaviors that involve human interactions, such as cards, and they are less likely to engage in gaming machines, which is more prevalent in Western cultures (Casino & Authority, 1999; Raylu & Oei, 2004).

Recent studies have indicated an increase in gambling behavior in Türkiye (Altıntaş et al., 2024; Aricak, 2019). However, the existing body of knowledge on GD is disproportionately derived from men from Western countries/cultures with non-clinical samples, which limits an understanding of clinical populations of women with GD in non-Western countries/cultures (Kairouz, Sowad, Lambo, Le Mesurier, & Nadeau, 2023; Mathieu, Barrault, Brunault, & Varescon, 2020). A recent expert study also emphasized the need for future research in non-Western countries/cultures (Czakó et al., 2025). Given the potential impact of traditional sociocultural norms on women's gambling behaviors and seeking of treatment, the limited sex-related data from non-Western countries/cultures represent a significant gap in both clinical and public health knowledge. Addressing this gap is important for informing national policy and designing specific interventions that may effectively meet the unique needs of women with GD.

### The present study

This study aimed to address an existing gap by examining sex-related differences in the sociodemographic and clinical characteristics of individuals diagnosed with GD and actively seeking treatment for GD in Türkiye. This study includes a unique nationwide clinical sample from Türkiye as a non-Western country/culture, comprising a substantial number of women, a demographic that is underrepresented in national and international GD studies. By identifying sex-related clinical features within the context of GD treatment in Türkiye, this study offers new insights that may help inform the development of more targeted and sex-sensitive prevention and intervention strategies in Türkiye.

## Methods

### Sample

The sample of the retrospective cross-sectional study consists of individuals who received psychosocial support for GD in one of 105 Green Crescent Counselling Centers (YEDAM) located in Türkiye and the Turkish Republic of Northern Cyprus (TRNC) between 2021 and 2024. The approach involved a retrospective analysis of information obtained from responses given by participants during face-to-face sessions with clinical psychologists at YEDAM, which were routinely recorded in the institutional electronic clinical management software (YEDAMSOFT). To be eligible for inclusion in the study, participants had to meet the following criteria: (a) have a clinical diagnosis of GD according to DSM-5 criteria (b) be 18 years of age or older (c) volunteer to participate in the study (d) have received psychosocial support for only GD at YEDAM (e) have given informed consent to participate in the study. Exclusion criteria included: (a) Having had applied for treatment of an addiction disorder other than GD, (b) having severe cognitive impairment that may have impaired the ability to respond to surveys, and (c) having missing response data on any of the key sociodemographic or clinical variables analyzed in the current study.

Between 2021 and 2024, an initial pool of 13,288 individuals registered for treatment at YEDAM. During the data extraction process, 94 individuals were excluded for seeking treatment for an addiction disorder other than GD, and 689 individuals were excluded due to missing response data on the key variables. After applying these exclusion criteria, a final eligible cohort of 12,505 individuals with GD was identified. To examine sex differences, participants were asked to indicate their sex in the study (12,175 men and 330 women). For the study, all women participants who met the inclusion criteria were included in the sample. Men were selected based on key demographic variables, such as age, marital status, and level of education, to create a comparable sample. Using this approach, 990-men participants were selected, resulting in a 1:3 ratio between women and men. This selection was conducted randomly, involving a combination of purposive and matched sampling strategies. The employed matching approach has been recommended for observational studies to reduce systematic group differences and enhance the reliability of comparisons between groups (Rosenbaum & Rubin, 1983; Stuart, 2010).

### Information about the treatment program

Founded in 1920, the Turkish Green Crescent Society is a non-governmental organization that has been addressing addictive disorders for 106 years and currently conducts prevention, advocacy, and rehabilitation activities related to alcohol, substances, tobacco, as well as behavioral addictions, including GD and gaming disorder. Operating within the framework of this society, YEDAM provides free outpatient psychosocial counseling services to individuals and their families seeking treatment for substance addictions such as those involving alcohol, tobacco, and other drugs, as well as behavioral addictions such as GD. Across the territory of Türkiye, there is at least one YEDAM center in each province, along with one center in the TRNC, for a total of 105 centers. The YEDAM treatment model uses cognitive-behavioral therapy, motivational interviewing techniques, and mindfulness-focused therapies in individual sessions. Individual treatment is supported through family sessions, group therapy, and workshops. Treatment usually involves weekly sessions and may vary in length depending on individual needs and treatment engagement, typically ranging between 8 and 12 sessions. Clinical psychologists and social workers who provide treatment at YEDAM complete intervention training specific to each type of addiction and then provide services in this field. Upon admission to YEDAM, individuals seeking treatment for GD undergo a routine, semi-structured initial assessment to gather comprehensive sociodemographic, clinical, behavioral, and social data. The present study exclusively utilizes baseline data obtained during this pre-treatment evaluation phase.

### Measurement

The clinical diagnosis of GD was established through comprehensive clinical interviews conducted by addiction-trained clinical psychologists during the initial assessment phase. During these interviews, the DSM-5 diagnostic criteria for GD were strictly applied, and the number of endorsed criteria was systematically recorded for each individual.

**Informed Consent Form:** Before the first session, participants were given a form stating that their data would be used for scientific research purposes, their identities would remain confidential, and that they could contact the researcher when desired or necessary.

**Sociodemographic and Clinical Data Form:** This form includes items assessing participant's sociodemographic characteristics and key clinical indicators related to GD. It contains demographic questions such as sex, marital status, educational level, and employment status. In addition, it assesses psychological characteristics, including histories of outpatient or inpatient psychological or psychiatric treatment, other types of addiction, and a history of suicide attempts or self-injury behavior. The form also includes GD-specific items, such as the age of gambling onset, the first type of gambling performed, preferred types of gambling activities (e.g., online casino games, sports betting, skill-based gambling), gambling behaviors among family and peers, and whether the participant has previously received treatment for GD. In addition, participants were asked to report their motivation to quit gambling during the initial assessment. The level of motivation to quit gambling was evaluated using a single Likert-type item: “*Are you considering quitting gambling?*” Responses were scored on a 5-point scale ranging from 0 (never) to 4 (always).

**Addiction Profile Index Clinical Form (API-C)**: The API-C consists of a three-point Likert scale and 21 items that assess factors such as psychological problems and personality features related to addictions. As the score on the scale increases, the likelihood of addiction-related mental health problems also increases. The scale is divided into six subscales: depression (4 items, e.g., “*When I thought about the future during the past year, I felt hopeless.*”), anxiety (3 items, e.g., “*When I thought about the future during the past year, I felt restless and anxious.*”), anger management problems (3 items, e.g., “*I experience uncontrollable outbursts of anger.*”), lack of assertiveness (5 items, e.g., “*I have difficulty expressing how I feel.*”), novelty-seeking behavior (3 items, e.g., “*I enjoy doing exciting or fun things, even if they are inappropriate or dangerous*”), and impulsivity (3 items, e.g., “*I have difficulty waiting to get what I want. I am impatient,*” and “*I cannot concentrate or concentrate easily.*”). These subscales demonstrate good internal consistency, with Cronbach's alpha values of 0.66, 0.75, 0.74, 0.70, 0.75, and 0.63, respectively, totaling 0.81 (Ögel, Koç, Başabak, İşmen, & Görücü, 2015). In the current sample, the subscales showed the following internal consistency coefficients: depression = 0.66, anxiety = 0.69, anger management problems = 0.72, lack of assertiveness = 0.56, novelty-seeking behavior = 0.74, and impulsivity = 0.52, with a total Cronbach's alpha of 0.80.

**Kocaeli Short Screening Scale for Psychological Trauma (Kocaeli-SHORT):** The scale, which was prepared based on the criteria for post-traumatic stress disorder (PTSD), is used to evaluate the symptoms of people who have experienced traumatic events. Traumatic stress reactions are evaluated as “present/absent” and consist of 4 items. The cut-off score is 3 and the Cronbach alpha coefficient is 0.625. In the current sample, the scale demonstrated good internal consistency, with a Cronbach's alpha of 0.79. As previously, those who received at least 3 points for the Kocaeli-SHORT were defined as at risk (Aker, Hamzaoğlu, & Boşgelmez, 2007).

### Statistical analyses

The SPSS version 29.0 package program was used for data analysis. Within the scope of descriptive statistics, qualitative variables were summarized with frequency and percentage distributions; quantitative variables were summarized with mean, standard deviation, median, minimum, and maximum values. The normality of data distribution was assessed using the Shapiro-Wilk test and Box-Plot graphs. Student's *t*-tests were used for comparisons of two independent groups with normal distributions; however, in cases where the normality assumption was violated, the Mann-Whitney *U* test was employed. Chi-square, Fisher's Exact test, and Fisher-Freeman-Halton tests were used for the comparison of qualitative variables. Specifically, Fisher's Exact test or the Fisher-Freeman-Halton test was applied when the expected minimum cell count in cross-tabulations was less than 5. To address potential confounding effects and to examine the moderating role of sex on gambling severity, a hierarchical multiple linear regression analysis was conducted with the number of endorsed DSM-5 criteria as the continuous dependent variable. In Step 1, sex, alongside key sociodemographic and clinical variables (age, duration of gambling, financial difficulties, depression, impulsivity, prior attempt to quit, gambling frequency, and preference for casino games), was entered to assess main effects. In Steps 2 and Step 3, interaction terms (Sex × Depression and Sex × Impulsivity, respectively) were added to test the moderating effects of sex. To prevent multicollinearity, all continuous predictor variables were mean centered before creating the interaction terms. Collinearity diagnostics were thoroughly assessed; all Variance Inflation Factor (VIF) values were well below the acceptable threshold of 5 (ranging from 1.02 to 1.81), indicating no multicollinearity issues.

In order to ensure balance between groups, a 1:3 matching was performed with the nearest neighbor method using R software (version 4.3.2) and the MatchIt package (version 4.5.1). Significance level was set at *p* < 0.05 in all statistical analyses.

### Ethics

The study procedures were carried out in accordance with the Declaration of Helsinki. Ethics committee approval was received for this study from Erciyes University (date: May 14, 2025; meeting number: 2025/254).

## Results

Descriptive and comparative analyses were conducted to examine sex-related differences in sociodemographic, gambling, and psychological characteristics among individuals seeking treatment for GD. Between 2021 and 2024, the final eligible dataset extracted from YEDAM comprised 12,505 individuals seeking treatment for GD. Between 2021 and 2024, most individuals seeking treatment for GD at YEDAM were men, although the proportion of women gradually increased over this period. In 2021, men constituted 98.5% of cases (1,595), while women accounted for 1.5% (25). In 2022, men continued to dominate with 98.1% (3,207 cases), and the proportion of women rose slightly to 1.9% (61). By 2023, the percentage of men decreased to 97.0% (3,703), whereas women represented 3.0% (113). In 2024, men comprised 96.6% (3,670 cases) and women 3.4% (131). Overall, while men consistently constituted most treatment seekers, the share of women numerically increased during this period.

Sociodemographic characteristics of individuals seeking treatment for GD were compared by sex (see Table 1). The employment rate of women was lower than that of men (75.5% vs 89.7%, *p* <.001). Accordingly, men were approximately 2.8 times more likely to be employed than women (OR = 2.82). Women were also more likely than men to experience gambling-related financial difficulties, regardless of any association with unemployment (*p*=.002; OR = 1.18).

**Table 1.** Comparison of sociodemographic characteristics of individuals seeking treatment for gambling disorder by sex

		Women (*n* = 330)		Men (*n* = 990)		Test statistic	*p*	Effect size (odds ratio/cohen's d)
		*n*	%	*n*	%			
Age, years	Mean ± SD	34.66 ± 9.61 (20–74)		34.66 ± 9.37 (20–70)		*t = −0.008*	^*b*^ *0.993*	d:0.008
Education	Primary education	6	1.8%	18	1.8%	*X*^*2*^ *= 0.010*	^*a*^ *0.995*	–
	Secondary education	127	38.5%	378	38.2%			
	University and above	197	59.7%	594	60.0%			
Employment status	Employed	249	75.5%	884	89.7%	*X*^*2*^ *= 41.647*	^*a*^ 0.001**	OR:2.819
	Unemployed	81	24.5%	102	10.3%			
Marital Status	Married	129	39.1%	392	39.6%	*X*^*2*^ *= 0.026*	^*a*^ *0.871*	OR:1.021
	Not married	201	60.9%	598	60.4%			
Lifetime alcohol or substance use	Alcohol	162	49.1%	470	47.5%	*X*^*2*^ *= 0.342*	^*a*^ *0.843*	–
	Substance	9	2.7%	31	3.1%			
	Never use	159	48.2%	489	49.4%			
Gambling-Related Financial Difficulties	No	133	40.3%	438	44.2%	*X*^*2*^ *= 17.237*	^*a*^ 0.002**	OR:1.175
	Yes	197	59.7%	552	55.8%			

*Note.* a = *Chi-Square Test*; b = *Student's t-test*; *p < 0.05 **p < 0.01.

The gambling-related characteristics of individuals seeking treatment for GD were compared by sex (see Table 2). Women reported a later age at gambling onset compared with men (*p* = .001). In terms of leisure time, women were more likely than men to report having substantial leisure time (*p*=.013; OR = 1.37). A history of non-gambling-related psychiatric or psychological treatment was more likely among women compared to men (*p*=.001; OR = 2.84). Women were also more likely than men to have made prior attempts to quit gambling (*p*=.027; OR = 4.92). Significant sex differences were also observed in gambling preferences. Most women preferred online casino games (81.2%), while men more frequently reported a preference for online sports betting (45.4%). Women were approximately 4.5 times more likely than men to prefer online casino games (OR = 4.47). Women were less likely than men to report having friends who gamble (*p*=.001; OR = 0.36). Finally, women reported a significantly later age of gambling onset (29.03 ± 9.19 years) compared with men (21.96 ± 7.70 years) (*t* = −13.74, *p* < .001).

**Table 2.** Sex-based comparison of gambling-related characteristics among individuals seeking treatment for gambling disorder

		Women (*n* = 330)		Men (*n* = 990)		Test statistic	*p*	Effect size (odds ratio/cohen's d)
		*n*	%	*n*	%			
Age of gambling onset, years	Mean ± SD	29.03 ± 9.19		21.96 ± 7.70		*t = −13.744*	^*b*^ 0.001**	d:−0.874
	<18	8	2.4%	322	32.5%			
	18–25	133	40.3%	429	43.3%			
	>25	189	57.3%	239	24.1%			
Amount of Leisure Time	Very limited	163	49.4%	567	57.3%	*X*^*2*^ *= 6.215*	^*a*^ 0.013*	OR:1.373
	Quite substantial	167	50.6%	423	42.7%			
History of Non-Gambling Psychiatric/Psychological Treatment	No	110	33.3%	581	58.7%	*X*^*2*^ *= 63.778*	^*a*^ 0.001**	OR:2.841
	Yes	220	66.7%	409	41.3%			
Prior Attempt to Quit Gambling	No	84	25.5%	195	19.7%	*X*^*2*^ *= 4.922*	^*a*^ 0.027*	OR:4.922
	Yes	246	74.5%	795	80.3%			
Prior GD Treatment (inpatient/outpatient)	No	246	74.5%	748	75.6%	*X*^*2*^ *= 0.136*	^*a*^ *0.713*	^-^
	Yes	84	25.5%	242	24.4%			
Gambling-Related Psychological Prior Treatment	No	232	75.8%	752	78.8%	*X*^*2*^ *= 1.226*	^*a*^ *0.268*	–
	Yes	74	24.2%	202	21.2%			
Gambling Preferences	Online sports betting	45	13.6%	449	45.4%	*X*^*2*^ *= 113.642*	^*c*^ 0.001**	OR:0.190
	Online casino games	268	81.2%	487	49.2%			OR:4.465
	Skill-based gambling (e.g., billiards, darts)	2	0.6%	17	1.7%			OR:0.275
	Speculative financial activities with gambling-like features (crypto etc)	4	1.2%	12	1.2%			OR:1.000
	Sports lottery, number lottery, scratch cards. national lottery	2	0.6%	4	0.4%			OR:1.503
	Other forms of gambling	9	2.7%	21	2.1%			OR:1.294
Family History of Gambling	No	208	63.0%	644	65.1%	*X*^*2*^ *= 0.441*	^*a*^ *0.506*	–
	Yes	122	37.0%	346	34.9%			
Gambling Behavior in Peer Group	No	149	45.2%	227	22.9%	*X*^*2*^ *= 59.998*	^*a*^ 0.001**	OR:0.361
	Yes	181	54.8%	763	77.1%			
Duration of Gambling (year)	Mean ± SD	5.63 ± 6.04		12.70 ± 8.81		*t = 53.424*	^*b*^ 0.001**	d:0.861
Frequency of Gambling (past week)	None	56	17.0%	161	16.3%	–	^*a*^ *0.343*	–
	Rarely	23	7.0%	80	8.1%			
	A few days a week	43	13.0%	158	16.0%			
	Most days of week	62	18.8%	209	21.1%			
	Almost every day	146	44.2%	382	38.6%			
Motivation for gambling abstinence	Mean ± SD	3.55 ± 0.82		3.55 ± 0.79		*t:−0.042*	^*b*^ *0.966*	d:0.008

*Note. N* = Sample size; a = *Chi-Square Test*; b = *Student's t-test*; c = Fisher-Freeman Halton Test; *p < 0.05 **p < 0.01.

The psychological characteristics of individuals seeking treatment for GD were compared by sex (see Table 3). According to DSM-5 diagnostic criteria, most women and men met the threshold for moderate to severe GD, with no sex-related difference observed in severity levels (*p*=.622). Women scored higher than men on the API-C subscales of depression, anxiety, and impulsivity (*p*=.001; *p*=.004). Additionally, women were more likely to score positive for being at risk for PTSD compared to men (*p*=.001; OR = 3.67). Women versus men were also more likely to report non-suicidal self-harm (*p*=.006; OR = 1.63) and suicidal ideation (*p*=.001; OR = 1.61).

**Table 3.** Sex-based comparison of addiction-related and mental health characteristics among individuals seeking treatment for gambling disorder

		Women (*n* = 330)		Men (*n* = 990)		Test statistic	*p*	Effect size (odds ratio/cohen's d)
		*n*	%	*n*	%			
Number of endorsed DSM-5 criteria		8.09 ± 1.27		7.96 ± 1.24		*Z:−2.522*	^*d*^ 0.012*	d:0.140
*Mild*		16	4.8%	55	5.6%	*X*^*2*^ *= 0.243*	^*a*^ *0.622*	–
Moderate/Severe		314	95.2%	935	94.4%			
API-C								
Depression		4.54 ± 1.89		3.87 ± 1.94		*t:−5.508*	^*b*^ 0.001**	d:−0.350
Anxiety		2.57 ± 1.76		1.92 ± 1.39		*t:−6.123*	^*b*^ 0.001**	d:−0.437
Anger management problems		2.18 ± 1.64		2.06 ± 1.61		*t:−1.181*	^*b*^ *0.238*	d:−0.075
Novelty-seeking behavior		1.55 ± 1.79		1.62 ± 1.72		*t:0.548*	^*b*^ *0.584*	d:0.035
Lack of assertiveness		3.05 ± 1.85		3.24 ± 1.93		*t:1.516*	^*b*^ *0.130*	d:0.096
Impulsivity		3.56 ± 1.52		3.24 ± 1.93		*t:−2.850*	^*b*^ 0.004**	d:−0.181
Possible Post Traumatic Stress Disorder (PTSD) (*N* = 446)	Risk not identified	98	72.6%	282	90.7%	*X*^*2*^ *= 24.413*	^*a*^ 0.001**	OR:3.671
	Risk identified	37	27.4%	29	9.3%			
Non-suicidal self-injury history	No	275	83.3%	882	89.1%	*X*^*2*^ *= 7.580*	^*a*^ 0.006**	OR:1.633
	Yes	55	16.7%	108	10.9%			
Suicidal ideation	No	171	51.8%	627	63.3%	*X*^*2*^ *= 13.727*	^*a*^ 0.001**	OR:1.606
	Yes	159	48.2%	363	36.7%			

*Note. N* = Sample size; a = *Chi-Square Test*; b = *Student's t-test*; c = Fisher-Freeman Halton Test; d = *Mann Whitney U Test;* API-C = Addiction Profile Index Clinical Form; PTSD = post-traumatic stress disorder; *p < 0.05 **p < 0.01.

A hierarchical multiple linear regression was conducted to predict DSM-5 GD severity, measured by the number of endorsed DSM-5 criteria (Table 4). The main effects model (Model 1) significantly predicted gambling severity, explaining 7.6% of the variance (*R*^2^ = .076). Controlling for sociodemographic and clinical variables, financial difficulties (*β* = .118, *p* < .001), depression (*β* = .198, *p* < .001), and gambling frequency (*β* = .067, *p* = .014) emerged as significant positive predictors. Notably, when these confounding factors were accounted for, sex was no longer a significant independent predictor of GD severity (*p* = .237).

**Table 4.** Hierarchical multiple linear regression analysis predicting DSM-5 gambling disorder severity

Predictor	Model 1		Model 2		Model 3	
	*β*	*p*	*β*	*p*	*β*	*p*
Sex (Women)	.036	.237	.039	.200	.040	.188
Age	−.047	.159	−.047	.160	−.048	.153
Duration of Gambling (year)	.067	.062	.067	.061	.066	.066
Gambling-Related Financial Difficulties	.118	<.001	.119	<.001	.119	<.001
Depression	.198	<.001	.209	<.001	.203	<.001
Impulsivity	.007	.813	.007	.820	.023	.494
Prior Attempt to Quit Gambling	.030	.269	.030	.262	.030	.257
Frequency of Gambling (past week)	.067	.014	.066	.015	.066	.014
Gambling Preference for Casino	.001	.979	.001	.975	−.001	.970
*Interaction Terms*						
Sex × Depression			−.022	.475	−.010	.757
Sex × Impulsivity					−.033	.314
*R* ^*2*^	.076		.076		.077	
ΔR^2^	.076		<.001		.001	
ΔF	11.90*		0.51		1.01	

*Note. Beta* = Standardized Coefficient. Continuous predictor variables were mean-centered. *p < .001.

To test for moderation, interaction terms for Sex × Depression and Sex × Impulsivity were added in Models 2 and 3. Neither the addition of these terms significantly improved the models (ΔR^2^ ≤ .001), nor were the specific interactions statistically significant (*p* = .475 and *p* = .314, respectively). This indicates that the exacerbating effects of depression and impulsivity on GD severity operate similarly across both men and women.

## Discussion

This is the first study in Türkiye to examine sex -related differences in GD using a large clinical sample that includes a substantial number of men and women actively seeking treatment for GD. Significant differences were observed between men and women in relation to certain gambling-related characteristics (e.g., gambling preferences, age at onset of gambling, duration of gambling) as well as mental health variables (e.g., depression, anxiety, impulsivity, PTSD, and suicidal thoughts). Although international studies have documented sex-related differences in gambling behavior and related clinical features in Western countries/cultures populations, research from non-Western countries/cultures remains limited, especially studies of women and sex -related differences. Drawing on a nationwide clinical dataset, this study provides novel and important insights into sex-specific patterns, particularly regarding women seeking treatment for GD in Türkiye.

The findings can be interpreted from multiple perspectives. The first pertains to sex differences in the proportions of men and women seeking treatment for GD. Consistent with prior research, the proportion of individuals seeking treatment for GD was predominantly men (Jiménez-Murcia et al., 2016; Salonen et al., 2022; Wejbera et al., 2021; Zakiniaeiz & Potenza, 2018). However, more recent evidence suggests an increase in GD among women, partly driven by changes in gambling environments, particularly the rapid expansion of online gambling platforms (Corney & Davis, 2010; López-Torres et al., 2021; McCarthy et al., 2019; Wardle, 2017). These platforms have made gambling more accessible and possibly less stigmatizing for women by removing physical and social barriers typically associated with traditional, men-dominated gambling venues. Our findings, which suggest that women seeking treatment for GD engage predominantly in online gambling and the increase in women seeking treatment over recent years, appear to reflect patterns observed in other jurisdictions. This finding is especially salient in the Türkiye context, where sociocultural norms have historically constrained women's gambling involvement by limiting access to physical gambling spaces. The growing availability of online gambling has shifted this dynamic, making gambling more accessible for women in Türkiye, and has likely contributed to the rise in women seeking treatment for GD.

A further consideration is the age at onset of gambling behavior and the time between onset and treatment-seeking in the sample. Prior research indicates that men are generally more likely than women to engage in gambling and tend to begin gambling at younger ages (Baker-Frampton, 2023; Carneiro et al., 2020; Díez, Aragay, Soms, Prat, & Casas, 2014; Gartner et al., 2022; Ronzitti et al., 2016). Factors such as socialization processes, greater risk-taking tendencies, and earlier exposure to gambling environments may underlie this pattern. In the present study, women reported a later average age at gambling onset compared to men. This discrepancy may stem from differences in initial exposure to specific types of gambling and the tendency for women to initiate gambling later in adulthood as a coping mechanism for emotional distress. Thus, women exhibited a shorter duration between age at gambling onset and seeking treatment, which may reflect the “telescoping effect” described in the literature, whereby women progress more rapidly from initial gambling to the development of problematic gambling (Grant et al., 2012). The extent to which this is linked to the type of gambling (e.g., online casino or isolated gambling) warrants further examination. Importantly, women reported experiencing greater financial difficulties related to their gambling compared to men. In the US, women, as compared to men, have reported greater financial stress in a general population sample (Armstrong, Ronzitti, Hoff, & Potenza, 2018). The extent to which GD-related financial difficulties in the current sample may be linked to specific factors, such as reduced employment rates among women, or the rapid progression of gambling severity that precipitates sudden financial crises, warrants further consideration and study. While lower employment rates among women may contribute to overall economic vulnerability, our results indicate that profound gambling-related financial difficulties in women are often observed irrespective of their employment status. Consequently, clinicians should look beyond employment status and specifically inquire about covert financial harms, such as hidden debts, borrowing from social circles, or the depletion of family savings. Taken together, these results suggest that men in Türkiye are more likely to start gambling earlier and persist for a longer period before seeking treatment. In contrast, women may transition more quickly to clinically significant gambling-related problems, prompting treatment-seeking and encounter heightened financial distress following the onset of gambling. As women are more likely to seek treatment than men for psychiatric concerns (Drapeau, Boyer, & Lesage, 2009), disentangling the progression of GD severity from treatment seeking will be important.

A third area worth examining is sex differences in potential reasons for gambling behavior. Previous studies have shown that men are more likely to gamble for reasons such as financial gain and sensation-seeking, whereas women tend to gamble to cope with emotional distress or manage negative emotions (Estévez et al., 2023; McCormack et al., 2014; Neophytou, Theodorou, Artemi, Theodorou, & Panayiotou, 2023; Walker & Shannon, 2006). In our study, women were significantly more likely to report having abundant free time. Although we did not examine gambling motivations between men and women, gambling among women may be linked to difficulties in managing leisure time (Echeburúa, González-Ortega, De Corral, & Polo-López, 2011; McCormack et al., 2014). In addition, the presence of gambling behavior among peers was found to be higher in men. One possible explanation for this may be men's greater susceptibility to peer pressure, which has been previously described among treatment-seeking individuals with GD (Echeburúa et al., 2011). Therefore, peer pressure may contribute to the occurrence of gambling behaviors. Alternate explanations (e.g., relating to the type of gambling, with sports gambling performed among groups of men friends and online casino games played in isolation) also warrant consideration.

An additional important dimension of sex differences lies in the types of gambling activities preferred by individuals. The existing literature suggests that men are more likely to engage in strategic forms of gambling, such as sports betting or poker, where elements of skill, competition, and control are perceived to play a role. In contrast, women have been shown to prefer non-strategic forms of gambling, such as slot machines or bingo, which are primarily chance-based and cognitively less demanding (Baenas et al., 2024; Echeburúa et al., 2011; Jiménez-Murcia et al., 2020; McCormack et al., 2014). Such patterns have been observed in US treatment-seeking and general population samples and among groups with gambling problems and recreational gambling, across age groups (Desai, Maciejewski, Pantalon, & Potenza, 2005, 2006; Potenza et al., 2001). These preferences may reflect underlying motivational differences; women may have more tendency to simpler, chance-based games as a means of emotional distraction or temporary relief from stress, whereas men may be more likely to be motivated by competition and financial reward. Consistent with this literature, the present study found that most women participants preferred online casino games, while men participants were more likely to prefer online sports betting. The greater occurrence of chance-based gambling among women in our sample may point to a tendency to avoid cognitively demanding or competitive environments, instead showing a preference for gambling formats that enable greater psychological disengagement. These sex-related gambling preferences and the underlying motivations should be considered when designing prevention and intervention strategies for GD.

Beyond gambling-related characteristics and preferences, co-occurring psychiatric concerns warrant attention, as they often influence clinical presentations, prognosis, and treatment outcomes of GD. Contrary to the previous study's findings involving clinical and general population samples, our study did not find that either men or women with GD reported comorbid substance use issues. However, consistent with prior research, women were more likely to report mood and anxiety disorders in relation to GD (Blanco, Hasin, Petry, Stinson, & Grant, 2006; Boughton & Falenchuk, 2007; Brand, Rodriguez‐Monguio, & Volberg, 2019; Estévez et al., 2023; González-Ortega, Echeburúa, de Corral, & Polo-López, 2014; Larsson & Håkansson, 2022; Miles, Rothschild, Åkesson, & Håkansson, 2023; Miller et al., 2023; Ronzitti et al., 2016). The high proportion of alcohol use in the sample was somewhat surprising given the social sanctions on alcohol consumption in Türkiye and suggests that GD may link to a broader range of less societally acceptable behaviors. The sex-related pattern may be understood with respect to sex-related gambling motivations: for men, gambling may constitute part of a broader repertoire of externalizing and risk-taking behaviors, whereas for women, it may serve as a coping strategy for psychological distress. The findings of the present study are consistent with this notion. Women reported significantly higher likelihoods of prior psychological or psychiatric treatment unrelated to gambling, suggesting a longer-standing history of emotional difficulties, greater treatment-seeking tendencies, or both. They also scored significantly higher on measures of depression, anxiety, and risk of PTSD. Furthermore, women reported increased odds of non-suicidal self-injury and suicidal ideation. Taken together, these results suggest that women may be more prone to engage in gambling as a maladaptive form of emotion regulation or escape from psychological distress, whereas men may incorporate gambling within a pattern of externalized behaviors centered on performance, control, or financial gain. Our findings strongly align with recent clinical observations in other European contexts. For instance, a recent study among Swedish treatment-seeking individuals with GD (Miller et al., 2023) reported an almost identical sex distribution: women were significantly overrepresented in the 'emotionally vulnerable' pathway with higher affective comorbidities, whereas men were more frequently categorized within the 'antisocial' pathway with concomitant substance use problems. This cross-cultural consistency reinforces the clinical utility of sex-sensitive assessments. These sex-related differences in co-occurring psychopathology emphasize the importance of clinical assessments that are sensitive to sex and treatment strategies tailored to the psychiatric concerns more frequent among women seeking treatment for GD.

An additional noteworthy and unexpected finding was that women with GD demonstrated higher levels of impulsivity compared to men. This finding differs from prior reports linking impulsivity more robustly to GD in men (Echeburúa et al., 2011, 2013; González-Ortega, Echeburúa, Corral, Polo-López, & Alberich, 2013; Lozano-Madrid et al., 2023; Shi & Li, 2023). Nonetheless, some prior studies have documented higher levels of impulsivity among women who gamble (Gooding et al., 2025; Lozano-Madrid et al., 2023). One possible explanation is that our sample consisted entirely of clinical cases actively seeking treatment, where impulsivity may emerge as a more salient characteristic among women, particularly in the context of co-occurring affective disorders and emotional dysregulation. The elevated impulsivity observed among women in our sample may be closely tied to their preference for non-strategic gambling formats, such as online casino games. Recent clinical evidence (Vintró-Alcaraz et al., 2022) demonstrated that non-strategic gamblers who are predominantly women exhibit significantly higher levels of emotion dysregulation and urgency (i.e., emotion-driven impulsivity) compared to strategic gamblers. Thus, the impulsivity seen in our women sample is likely a maladaptive, emotion-driven dysregulation utilized to cope with negative affective states, rather than traditional sensation-seeking. In this regard, elevated impulsivity may also contribute to the telescoping effect, whereby women progress more rapidly from gambling initiation to disorder and treatment-seeking. These findings raise the possibility that impulsivity may be regarded as a sex-sensitive clinical marker with direct implications for treatment planning. In line with this perspective, women in Türkiye seeking treatment for GD may particularly benefit from more individualized treatment strategies targeting impulse control through a combination of pharmacological interventions, cognitive-behavioral therapy, family involvement, and supportive group-based approaches (Lozano-Madrid et al., 2023).

Importantly, to directly address potential confounding factors, our multivariate regression analysis provided a crucial nuance to the observed sex differences. When controlling for clinical and sociodemographic variables, financial difficulties, depression, and gambling frequency emerged as robust independent predictors of GD severity, whereas participant's sex lost its independent predictive value. This finding aligns with the 'proxy effect' hypothesis (Nelson, LaPlante, LaBrie, & Shaffer, 2006) and is further supported by a recent comprehensive review (Gartner et al., 2022), which suggests that gender and sex differences in GD progression are often artifacts of underlying psychosocial vulnerabilities. Furthermore, our lack of significant moderation effects (Sex × Depression and Sex × Impulsivity) indicates that once severe psychological distress or impulsivity is present, it exacerbates GD severity equally for both men and women. Thus, while women in our sample presented with higher rates of depression and impulsivity, the detrimental mechanisms of these factors on GD severity operate universally across sexes.

### Strengths and limitations

This study has several strengths. First, it fills an important gap in the Turkish and broader non-Western countries/cultures literature on GD by examining sex-based differences among treatment-seeking individuals, a subject that remains underexplored despite the rising prevalence of gambling behavior in Türkiye (Altıntaş et al., 2024; Aricak, 2019). Secondly, the study includes a substantial proportion of women, a group that is underrepresented in national and international GD research. Considering the influence of traditional sociocultural norms on women's gambling behaviors and help-seeking tendencies, this focus allows for the identification of sex-specific clinical patterns within a culturally unique context. Thirdly, the use of a large nationwide clinical dataset enhances the reliability, generalizability, and clinical relevance of the findings, as previous studies have mostly relied on non-clinical samples. By providing some of the first empirical insights into the nature of GD among Turkish women seeking treatment for GD, the study establishes a foundation for developing more targeted, sex -sensitive prevention and intervention strategies, as well as informing national mental health policies.

This study has several limitations that warrant consideration. Its retrospective and cross-sectional design limits causal inferences regarding the observed sex-related differences in gambling behaviors and co-occurring psychiatric concerns. Reliance on self-reported data may have introduced recall or reporting bias, particularly in a sociocultural context where gambling and mental health problems are highly stigmatized. Although matched sampling enhanced comparability between women and men, residual confounding from unmeasured variables (e.g., socioeconomic status, trauma history) cannot be excluded. Moreover, the findings are restricted to treatment-seeking individuals presenting at YEDAM centers, thereby limiting generalizability to the broader population of individuals with GD in Türkiye who do not access formal treatment. Finally, the absence of longitudinal data precludes examination of the temporal course of GD and potential sex-specific treatment outcomes over time, which may vary by type of treatment received.

Furthermore, the characteristics of our sample are inherently influenced by the specific treatment setting. In Türkiye, YEDAM is unique as a nationwide, free-of-charge center providing strictly confidential, structured, multidimensional psychosocial rehabilitation for GD. Other treatment options are largely confined to private hospitals and a limited number of state-run Alcohol and Substance Addiction Treatment and Training Centres (AMATEM), both of which typically focus on pharmacotherapy. Consequently, individuals with active and severe concurrent substance use disorders, severe psychiatric comorbidities, or legal/probation mandates are typically initially directed to AMATEM units. Although AMATEMs frequently refer these complex cases to YEDAM for subsequent psychosocial rehabilitation, a subset of referred individuals often experience attrition during this transition of care. Thus, these complex cases may remain underrepresented in our sample.

## Conclusion

This nationwide study, the first to systematically examine sex-related differences in GD in Türkiye, addresses an important knowledge gap by drawing on a large clinical dataset of treatment-seeking individuals, including a substantial number of women. The findings reveal distinct clinical profiles: women exhibited later onset but more rapid progression to treatment (suggesting a telescoping trajectory), greater self-reported financial difficulties, higher impulsivity, depression, anxiety, and PTSD scores. The results suggest a need for sex-sensitive assessment and tailored interventions that specifically target emotion regulation difficulties and impulsivity in Turkish women seeking treatment for GD, while also examining further and accounting for cultural contexts. Specifically, given the high preference for online casino games and elevated rates of PTSD and depression among women in this sample, future treatment protocols in Türkiye should integrate trauma-informed care and targeted emotion regulation modules. By focusing on treatment-seeking cases, this study provides clinically grounded insights into the manifestation of GD and suggests the relevance of developing culturally informed, sex-responsive prevention and treatment strategies to more effectively improve outcomes.

## Data Availability

The data that support the findings of this study are available from the corresponding author, upon reasonable request.
